# Pediatric patients with mesenteric cystic lymphangioma: A case series

**DOI:** 10.1016/j.ijscr.2019.09.034

**Published:** 2019-10-01

**Authors:** Gunadi, Gibran Kashogi, Dedi Prasetya, Aditya Rifqi Fauzi, Eddy Daryanto, Andi Dwihantoro

**Affiliations:** a Pediatric Surgery Division, Department of Surgery, Faculty of Medicine, UGM/Dr. Sardjito Hospital, Yogyakarta 55281, Indonesia; b Pediatric Surgery Division, Department of Surgery, PKU Muhammadiyah Wonosobo Hospital, Wonosobo 56371, Indonesia; c Pediatric Surgery Division, Department of Surgery, Banyumas General Hospital, Banyumas 53192, Indonesia

**Keywords:** Acute abdomen, Asymptomatic abdominal mass, Mesenteric cystic lymphangioma, Radiologic findings

## Abstract

**Introduction:**

Mesenteric cystic lymphangioma (MCL) is a benign malformation of the lymphatic system. MCL seldomly occurs with a very rare incidence of around 1: 250,000. This neoplasm is more common in pediatric patients. Symptoms can appear with various clinical features ranging from asymptomatic abdominal mass to acute abdomen.

**Presentation of cases:**

Here we report four pediatric patients with MCL: two males presented with bilious vomiting and ileus, one female with abdominal pain, and one female with asymptomatic abdominal mass. All patients underwent explorative laparotomy and mass excision was done and/or marsupialization. All patients were discharged at the third – fourth postoperative day uneventfully.

**Discussion:**

Although benign, lymphangioma can cause other symptoms such as bleeding, torsion, or lymphangioma rupture. Therefore, MCL should be considered as one of the differential diagnoses in acute abdominal and abdominal mass cases in children.

**Conclusion:**

In conclusion, although sometimes asymptomatic and found incidentally, the MCL should be resected because it has the potential to grow and invade vital organs and cause life-threatening complications.

## Highlights

• MCL symptoms and signs may occur with various clinical features, from asymptomatic abdominal mass to acute abdomen.
• MCL should be considered as one of the differential diagnoses in acute abdomen and intraabdominal tumor cases in children.
• MCL has potential to grow and invade vital organs, therefore, resection/marsupialization is preferable for its management.

## 1 Introduction

Mesenteric cystic lymphangioma (MCL) is a benign neoplasm originating from the lymphatic system. This tumor is very rare with an incidence of 1: 250,000 and is commonly found in children, of which 60% appear at birth, and 40% by one year of age. MCL represent 5–6% of benign tumors in children [1,2].

The predilection of tumors is in the head and neck (70%), axillary (20%), and internal organs (10%) [3]. Diagnosis is confirmed by histopathological findings of lymphatic vessels restricted to the connective tissue of endothelial cells and smooth muscle tissues [2].

The main therapy for MCL is mass resection with an open explorative laparotomy or laparoscopy approach. In some cases, MCL that is tightly attached to the intestinal wall might need an intestinal resection [2,4]. In this study, we reported four patients with MCL: two males presented with bilious vomiting and ileus, one female with abdominal pain, and one female with asymptomatic abdominal mass. This research work has been reported in line with PROCESS criteria [5].

## 2 Presentation of cases

### 2.1 Case 1

**Fig. 1:**
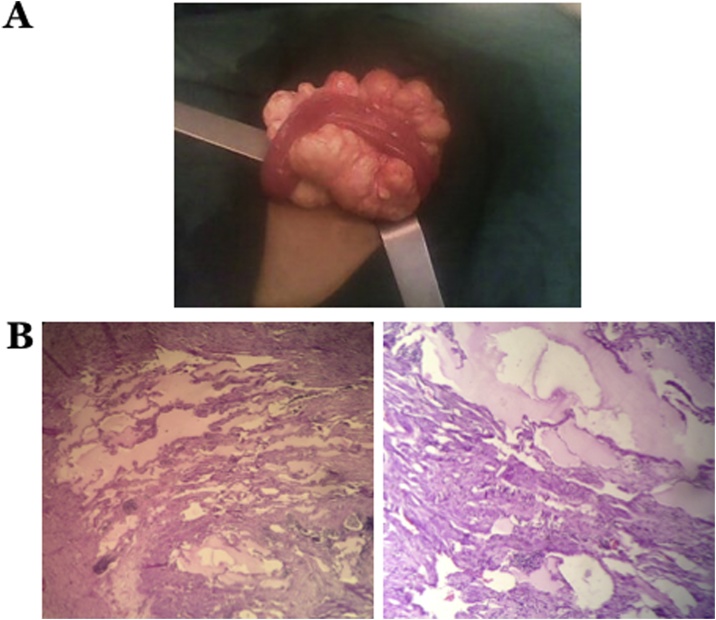
A) Intraoperative findings of MCL showing thin-walled chylous fluid-filled, significantly attached to the jejunum. B) Tissue samples were stained using hematoxylin and eosin. Histopathological view at 40× (left) and 100× (right) magnification showed the dilated cystic spaces, coated with endothelial, which contained amorphous eosinophilic fluid, supporting the diagnosis of lymphangioma.

A 3-year-old male presented to the emergency room with chief complaint of profuse bilious vomiting of more than ten times per day. Physical examination showed a palpable mass on the periumbilical region, measuring around 8 cm, mobile, and accompanied by tenderness. Abdominal X-ray was performed, and no sign of ileus was found, whereas intra-abdominal mass was found on ultrasonography. The patient then underwent an explorative laparotomy. A yellowish-white tumor mass containing chylous fluid was found in the jejunal mesentery measuring 15 × 8 × 5 cm^3^ that pressed against the jejunum (Type 2 MCL) (Fig. 1A). The tumor was located 15 cm from the Treitz ligament. Next, we excised the tumor mass except the one significantly attached to the jejunum, and sent the tissue samples for histopathological examination (Fig. 1B). Postoperatively, patients got broad spectrum antibiotics, metronidazole, and analgesics accordingly. The patient was discharged uneventfully at the fourth postoperative day (POD4).

### 2.2 Case 2

**Fig. 2:**
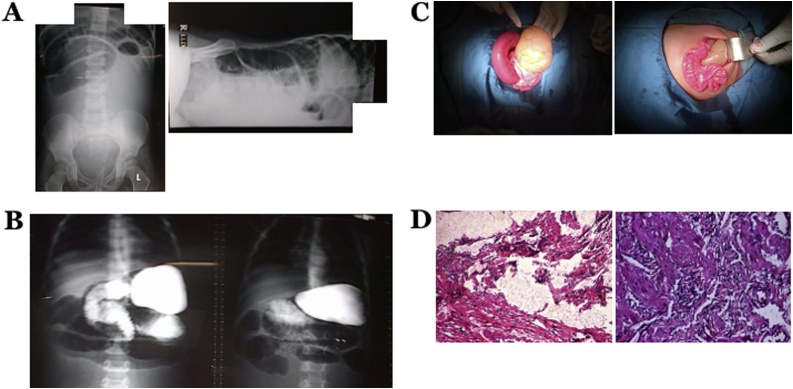
A) Abdominal X-ray showed small bowel distention. B) Barium follow-through showing volvulus with very high semi-organoaxial obstruction at the jejunum. C) Intraoperative findings showed thin-walled MCL containing serous fluid. D) Tissue samples were stained using Hematoxylin-eosin. Histopathological view at 40× (left) and 100× (right) magnification showed tissue cyst wall in the form of connective tissue and smooth muscle infiltration by lymphocytes and blood vessels dilated portion and extravasation of erythrocytes supporting a diagnosis of cystic lymphangioma with inflammation.

A 4-year-old male was referred to our hospital for having an ileus. The patient’s complaints were bilious vomiting accompanied by bloating and abdominal pain for four days. The patient had a history of intermittent abdominal pain in the last one year before admission. On physical examination, abdominal distension was found. Abdominal X-ray was performed and showed small bowel obstruction (Fig. 2A), whereas the barium follow-through findings showed volvulus with very high semi-organoaxial obstruction at the jejunum (Fig. 2B). The patient underwent exploratory laparotomy and two mesenteric cysts were found with a diameter of each around 10 cm and 8 cm (Fig. 2C). Both cysts were located at 15 cm and 10 cm from the Treitz ligament. We performed an incision to drain the fluid-filled cysts. Marsupialization and partial excision were performed; tissue samples were sent for histopathological examination and the results supported the diagnosis of cystic lymphangioma (Type 1 MCL) (Fig. 2D). Postoperatively, the patient received broad spectrum antibiotics, metronidazole, and analgesics appropriately. The patient was discharged at the POD4.

### 2.3 Case 3

**Fig. 3:**
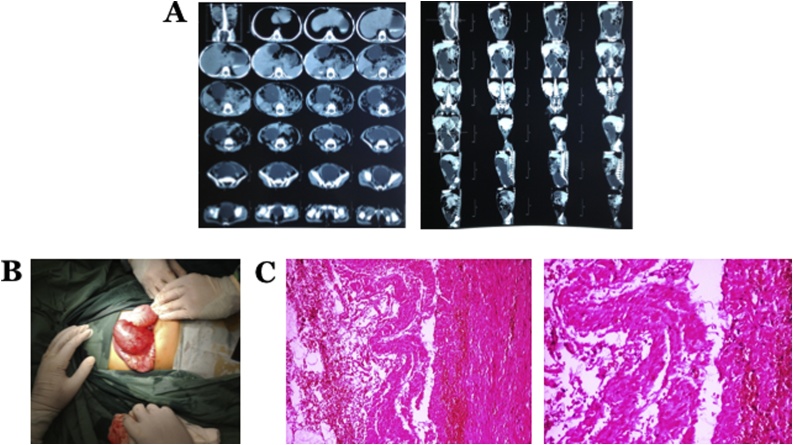
A) Abdominal CT scan showed oval tubular cystic mass in the middle of the abdomen which extends into the pelvic cavity and displaces the bowel to the anterior; tumor origin difficult to evaluate. B) Intraoperative findings showed thin-walled cystic lymphangioma contains serous fluid. C) Tissue samples were stained using hematoxylin and eosin. Histopathological view at 40× (left) and 100× (right) magnification showed the dilated cystic spaces, coated with endothelial, contained amorphous eosinophilic fluid, supporting the diagnosis of lymphangioma.

A 3-year-old girl was referred to our hospital because of intra-abdominal tumors. Patient complained of a mass becoming bigger in the lower abdomen since the last three months accompanied by weight loss. On physical examination, abdominal distension was found with a palpable mass in the epigastric to the suprapubic area. Computerized tomography scans were performed and showed an oval tubular mass in the middle of the abdomen (Fig. 3A). The patient underwent explorative laparotomy, and we a found tubular-shaped MCL sized around 10 × 5 cm that had entered the retroperitoneal and pelvic cavity (Fig. 3B). We performed cyst incision to drain the fluid. Marsupialization and partial excision of the mass were performed; tissue samples were sent for histopathological examination (Fig. 3C). Postoperative diagnosis for this patient was Type 4 MCL. Postoperatively, the patient received broad spectrum antibiotic, metronidazole, and analgesic accordingly. The patient was discharged at the POD4.

### 2.4 Case 4

**Fig. 4:**
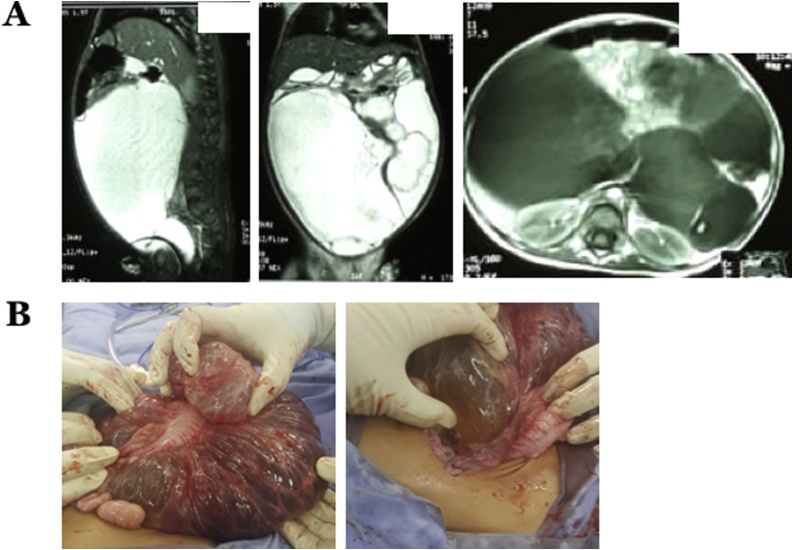
A) Contrast magnetic resonance imaging revealed suspicious multilocular large mesenteric cysts. B) Intraoperative findings showed multiple mesenteric cysts attached to the transverse colon and descending colon.

A 19 months old girl was referred to our hospital because of intraabdominal tumors that were enlarging since the patient was an infant. The patient’s complaints were bilious vomiting accompanied by abdominal pain for seven days before admission. On physical examination, abdominal distension was found with a mobile palpable cystic mass, almost encompassing the entire abdomen region. Abdominal X-ray was performed showing enlarged liver and enlarged kidney, whereas the contrast Magnetic Resonance Imagery showed suspiciously large mesenteric cysts, which were multilocular (Fig. 4A). The patient underwent exploratory laparotomy and multiple mesenteric cysts were found, multilocular with the largest sized around 20 × 15 × 10 cm^3^ that had entered the retroperitoneal area and attached in part to the transverse colon and descending colon (Type 4 MCL) (Fig. 4B). We performed cyst incision to drain the fluid. Marsupialization and partial excision of the mass were performed; tissue samples were sent for histopathological examination. The patient received broad spectrum antibiotics and analgesics post operatively and was discharged at the POD3.

## 3 Discussion

Here, we reported four MCL cases with various clinical manifestations. Clinical features of MCL can vary, with the predominance of asymptomatic abdominal masses, but if complications arise, they can manifest as acute abdomen (volvulus or intestinal obstruction), depending on the size and location of the mass. Although benign, lymphangioma can cause other symptoms such as bleeding, torsion, or lymphangioma rupture. Therefore, MCL should be considered as one of the differential diagnoses in acute abdominal cases in children [2].

MCL might be classified into four types as follows [3]: Type 1) Pedicled MCL, which can cause intestinal volvulus, torsion, and necrosis or rapidly growing masses. This type can usually be resected without the risk of injuring intestinal blood flow. Type 2) Sessile MCL, with the predilection on the mesentery border. This type is less mobile than Type 1. Bowel resection can interfere with blood flow and require intestinal resection. Type 3) MCL with retroperitoneal extension, where the involvement of vital retroperitoneal structures such as the aorta and vena cava can cause complete mass resection to be impossible. Type 4) Multicentric MCL, for which the prognosis of this type is doubtful in cases of extensive intraabdominal and retroperitoneal organ involvement. In this case series, our patients had Type 1 (1 case), Type 2 (1 case), and Type 4 (2 cases) and all underwent explorative laparotomy with uneventful recovery within 3–4 days postoperatively.

Some patients with lymphangiomas are reported to experience spontaneous regression. Drainage can be an alternative therapy for intraabdominal lymphangioma in patients at high risk but this procedure has a high risk of recurrence and the risk of perforation during drainage [6]. Partial resection and marsupialization might also have risks such as bleeding, infection, and lymphatic fistula [4], however, our patients did not show such complications.

## 4 Conclusion

Although asymptomatic and discovered incidentally, MCL needs to be resected because of the potential for growth and invasion of vital organs that can cause life-threatening complications. MCL total resection with clear microscopic boundary was the best approach if the facility provides the necessary supports.

## Funding

No funding was received for this study.

## Ethical approval

This study has been approved by the Ethical Committee of Faculty of Medicine, Public Health and Nursing, Universitas Gadjah Mada/Dr. Sardjito Hospital (KE/FK/0569/EC/2019).

## Consent

Written informed consent was obtained from the patients’ parents for publication of this case report and accompanying images. A copy of the written consent is available for review by the Editor-in-Chief of this journal on reasonable request.

## Author’s contribution

Gunadi conceived the study and writing the manuscript. Gibran Kashogi and Dedi Prasetya collected the data. Aditya Rifqi Fauzi, Eddy Daryanto, and Andi Dwihantoro critically revised the manuscript for intellectual content. Gunadi, Gibran Kashogi, Dedi Prasetya, Eddy Daryanto, and Andi Dwihantoro facilitated all project-related task.

## Registration of research studies

researchregistry5084.

## Guarantor

Gunadi.

## Provenance and peer review

Not commissioned, externally peer-reviewed.

## CRediT authorship contribution statement

Conceptualization, Investigation, Methodology, Supervision, Validation, Writing - review & editing. **Gibran Kashogi:** Data curation. **Dedi Prasetya:** Data curation, Resources. **Aditya Rifqi Fauzi:** Writing - original draft. **Eddy Daryanto:** Data curation, Resources. **Andi Dwihantoro:** Data curation, Resources.

## Declaration of Competing Interest

No potential conflict of interest relevant to this article was reported.
